# Parents' responses to receiving sickle cell or cystic fibrosis carrier results for their child following newborn screening

**DOI:** 10.1038/ejhg.2014.126

**Published:** 2014-07-09

**Authors:** Fiona Ulph, Tim Cullinan, Nadeem Qureshi, Joe Kai

**Affiliations:** 1 University of Manchester, Manchester, UK; 2 East Staffordshire Clinical Commissioning Group, Burton-on-Trent, UK; 3 University of Nottingham, Nottingham, UK

## Abstract

Universal newborn screening for sickle cell disorders and cystic fibrosis aims to enable the early identification and treatment of affected babies. Screening can also identify infants who are healthy carriers, with carrier results being the commonest outcome for parents and professionals to discuss in practice. However it is unclear what the effect will be on parents on being informed of their baby's carrier result. Semi-structured face-to-face interviews were conducted with a purposeful sample of 67 family members (49 mothers, 16 fathers, 2 grandparents) of 51 infants identified by universal newborn screening as carriers of cystic fibrosis (*n*=27) and sickle cell (*n*=24), across all health regions in England. Data were analysed by thematic analysis with subsequent respondent validation. Untoward anxiety or distress among parents appeared influenced by how results were conveyed, rather than the carrier result *per se.* Parents who had more prior awareness of carrier status or the possibility of a carrier result assimilated the information more readily. Being left in an information vacuum while awaiting results, or before seeing a professional, led some parents to fear that their child had a serious health condition. Parental distress and anxiety appeared mostly transient, subsiding with understanding of carrier status and communication with a professional. Parents regarded carrier results as valuable information and sought to share this with their families and to inform their children in the future. However parents needed greater support after communication of results in considering and accessing cascade testing, and negotiating further communication within their families.

## Introduction

Newborn bloodspot screening is regarded as a significant public health achievement in the developed world.^1^ Newborn screening (NBS) policies vary internationally in terms of diseases screened for, with differing approaches to consent.^1^ For example, newborn screening is usually mandatory in the US, whereas in Canada a largely ‘opt-out' policy requires testing for all newborns unless parents decline testing. NBS for cystic fibrosis (CF) and sickle cell diseases (SCD) was implemented nationally across the whole of England in 2007 but is offered to parents of all newborns using a voluntary informed consent model to ‘opt in' to screening.^2^ Screening guidelines and training materials stress the central role that information provision has in parents ability to consent.^3, 4^ Yet, evidence suggests several barriers to this communication including parents failing to appreciate the personal relevance of screening information,^5^ information overload during pregnancy^6^ and conflicting familial or existing personal knowledge.^7^ The challenge of creating antenatal communication protocols which meet parents' information needs, provided when parents are able to assimilate the information and appreciate the personal relevance is recognised.^8^

Although the rationale for informed consent is based on respecting parents' autonomy,^9^ information provision may influence how parents respond to NBS results. In relation to metabolic disorders, NBS carrier or false positive results can trigger anxiety if misunderstood, or if parents with children undergoing further testing related to false positive results are not provided with timely and adequate information.^10, 11^ Such anxiety has been linked to impaired relationships with the baby^11, 12, 13^ and lasting effects of stress during childhood.^14^ This suggests that earlier fears that newborn PKU screening may trigger the vulnerable child syndrome for some parents^15, 16^ may have been realised. For these parents multiple and/or specialist health professional consultations are often needed to allay such anxiety.^17^ Research in the US has suggested that inadequate preparation for NBS results can exacerbate anxiety and shock for parents who are informed that their child is a genetic carrier of cystic fibrosis or sickle cell^18^ and that the content of information given can affect parents' reactions.^19^ This paper reports a qualitative exploration of the impact of receiving cystic fibrosis or sickle cell carrier results on parents following screening of their newborns in England, where the use of an informed consent model could potentially reduce possible harms of disclosing carrier status.^20, 21, 22^ This study formed a part of wider cross-sectional research in the practice, methods and experience of communication following newborn screening across England.^6^

## Materials and Methods

### Recruitment

Recruitment sites, and parents informed of their baby's carrier results were purposively sampled to ensure the wide range of differing methods of communicating carrier status information in England were captured.^21^ Parents were recruited from across all nine health regions in England in 2008. Health professionals distributed study packs to parents who had received newborn carrier results. Parents who returned ‘consent to contact' forms were contacted by a researcher to discuss the study, and arrange an interview if appropriate, with informed consent undertaken at interview. Parents who returned the translation request form were contacted with a professional interpreter and a three-way telephone conversation was held to discuss participation. Following the interviews, parents were informed that they would receive a research summary, and asked if they were willing to be contacted again for discussion and validation of preliminary findings.

### Data generation

Semi-structured interviews were conducted in participants' homes, using professional interpreters where necessary for non-English speakers. Parents were encouraged to relate their experiences from when they were first aware that their child was to be screened and interviews flexibly followed a topic guide which was modified following initial interviews. Interviews were recorded and transcribed verbatim in English. Interview transcripts translated from other languages were checked by independent interpreters for accuracy and equivalence of meaning.

### Analysis and validation

Data generation and analysis were iterative, with each informing the other using techniques derived from grounded theory. This was undertaken by field researchers with health psychology and social science backgrounds, with the wider investigating team (clinical primary care/genetics academics) contributing to development of themes from a multidisciplinary perspective. Data were managed within NVivo software. Further theoretical sampling and data collection sought deviant cases to extend and challenge the analysis until saturation was achieved. This was followed by a respondent validation exercise in which all participants were sent a summary of the findings, translated where appropriate. Participants were then invited to participate in semi-structured telephone interviews to comment on the findings, reflect further on their experience and add any further views.

#### Ethical approval

An NHS multicentre research ethics committee (West Midlands) granted ethical approval to the study.

## Results

Interviews were conducted with 67 family members of 51 infants identified by newborn screening as carriers of CF (27) and SC (24). Interviews were conducted with parents across all the nine health regions of England, including high and low prevalence areas for sickle cell disorders. Eight interviews were conducted with professional interpreters (French=3; Bengali=3; Portuguese=2). Further semi-structured telephone interviews, for respondent validation, were conducted with 17 parents, three to fourteen months after their initial interview. Characteristics of participants and their infants are summarised in Tables 1 and 2.

Parents in the sample experienced a wide range of methods of communication of carrier results (see Supplementary Tables 3 and 4).

Four principal aspects of receiving a carrier result following newborn screening which affected parents were developed from data analysis: the impact of knowing the carrier result; effect of the process of communication; cascade testing within the family; and sharing of carrier information with extended family.

### Impact of knowing carrier result

With the exception of one parent who was uncertain, all parents felt it was important that they be informed of their child's carrier result. This was regarded as valuable new information about and for their child, gained fortuitously.

*I am happy to know it, because it didn't involve any additional tests you know which is always nice for a little baby and it's, I think, it is valid information.*[…] *I could even argue that there is a need to know...***(#33, mother of an SC carrier; low SC prevalence area)**.

Most parents were not concerned or distressed by the carrier result *per se*. Most recognised that this was not ‘bad news' and it had no direct adverse effect on their child.

*I don't think* [parents] *should be upset about this, it is very important not just for them, but for the child involved as well.* (**#3, Mother of an SC carrier; high SC prevalence area.)**

Some parents felt positively reassured when they heard that their child was a carrier, particularly those parents awaiting the results of a second IRT blood test for CF ), who had worried about their child being affected by the condition. (The CF newborn screening protocol in England aims to identify a maximum of children with CF, while minimising carrier detection. This involves measuring immunoreactive trypsinogen (IRT) levels to identify babies with levels exceeding the 99.5 percentile, indicative of CF. Samples from newborns with raised IRT levels undergo DNA analysis to establish if the child has two mutations, and is therefore likely to have CF, or has one mutation and is probably a carrier. Children with suspected CF are referred for a clinical diagnosis. Those with one mutation, or an initial IRT >99.9 percentile and no detected mutation, require a second bloodspot test to verify if IRT is still elevated at 3–4 weeks when IRT levels are more discriminatory. In those children with one mutation, where the IRT remains elevated the likelihood of CF is regarded as high and triggers clinical referral, whereas if not still elevated the likelihood of CF is low and the child is regarded a healthy carrier.)

*When they said she didn't have it* [affected with CF] *I was ‘away', that was it, I'd closed down, that was fine* […] *for me it was like ‘I can switch off now'.* (**#37, Mother of a CF carrier.)**

Some parents underlined how they felt identification of carriers, and being informed of this, was helpful.

[…] *people knowing that they've got this status is really important, because there might be less people with the sickle cell disease in the future for people having that knowledge.* […] *hopefully the majority will take it on board and think ‘I don't want my child to be ill and so therefore I'm going to be careful' you know and that's surely important.***(#35, Mother of an SC carrier; low prevalence area.)**

When asked to reflect on the possibility that technology might ensure that carriers were not identified via NBS, parents did not support this:

*If you stop telling people that they're carriers, that's got to be going in the wrong direction because at least if you're a carrier you know before she has children hopefully they'll talk about it and he'll go and get tested so they know what the likelihood …*(**#37, mother of a CF carrier, first child)**.

Although most parents appeared to understand the ‘benign' implications of their baby's carrier status, a minority of parents (four families, two of SC carriers, two of CF carriers) remained unduly concerned about their child's health after receiving results. They expressed negative or ambivalent reactions to knowledge of their child's carrier result such as guilt or had prominent concerns about the child's health.

*So I still worry and I just think ‘She's still little, anything she gets now could bring something else out' you know you've still got it in your head even though it's been cleared up for you, you still worry a little bit that how can something genetically just be as black and white as that?***(#40 mother CF carrier)**.

### Effect of process of communication

Some parents experienced distress or anxiety linked to their experience of newborn screening, in particular for CF (12/27 CF carrier families, 2/24 SC carrier families). This ranged from mild, short-lived anxiety, to more profound distress. This could manifest in reducing the child's interaction with others, arguments between couples, alteration of life plans and an inability to conduct tasks of daily living such as going to work or socialising. Most parents who reported such distress reflected that how carrier result information had been communicated to them had been the cause ‘… to some extent these things are often not so much about what you tell me but how you tell me' (**#55, mother of an SC carrier**). This was particularly salient in relation to communication around the time of the repeat blood sample in CF screening. (The CF newborn screening protocol in England aims to identify a maximum of children with CF, while minimising carrier detection. This involves measuring immunoreactive trypsinogen (IRT) levels to identify babies with levels exceeding the 99.5 percentile, indicative of CF. Samples from newborns with raised IRT levels undergo DNA analysis to establish if the child has two mutations, and is therefore likely to have CF, or has one mutation and is probably a carrier. Children with suspected CF are referred for a clinical diagnosis. Those with one mutation, or an initial IRT >99.9 percentile and no detected mutation, require a second bloodspot test to verify if IRT is still elevated at 3–4 weeks when IRT levels are more discriminatory. In those children with one mutation, where the IRT remains elevated the likelihood of CF is regarded as high and triggers clinical referral, whereas if not still elevated the likelihood of CF is low and the child is regarded a healthy carrier.)

**Mother:***I really wanted to get across just how really bad the process was for us, but then the relief of eventually getting the results and the relief of knowing that it wasn't cystic fibrosis and the problems of looking to know that we're a carrier and that our baby's going to be absolutely fine really. It was more the process of the actual results you know I think I probably had the hardest time dealing with it because it seemed to be never-ending.*

**Father:***It's the length of time, the lack of communication, lack of knowledge.***(#50, Parents of a CF carrier; told would hear screening result within 4 weeks, but contacted 6 weeks later, on a Sunday, by a midwife saying ‘problem with heel prick test' and their child retested that day at a weekend. Told poor sample, with not enough blood. Second sample was then lost.)**

Poor communication that failed to anticipate or address concerns appeared to have a major impact at this time. This included examples of parents of carriers who had become concerned that their child was chronically ill or perceived their child as fragile and so limited their interaction with others.

*You think your daughter's seriously ill and could die and will need physiotherapy all right through her life; will never be able to integrate properly at school and you're thinking ‘Well, what's going to happen career-wise?' because you plan that she'll go to full-time nursery and actually she won't be able to do that, so maybe we'll have to give up work, […] so your mind just goes on, and on, and on.***(#4, Father of a CF carrier; parents had been told that if they heard nothing in 2 weeks then ‘everything was ok' in the 4th week, got a phone message stating that there was a 'problem with heel prick test'. When parents called the contact number, they were told the CF test was positive and that it appeared their daughter may have CF, but the service was unable to give parents further information at that stage.)**

The data suggested that if parents were left in limbo regarding their child's result at any stage, it could lead to rumination and catastrophic thinking. Others simply felt unable to deal with people's enquiries about the results while waiting:

[...] *I mean it literally reached a point where I said ‘I don't want to see anybody, I want you to leave* [name of child] *and me on our own and not have to …. ' Because every time you're speaking to someone as well, that did know our situation, it would always come back to ‘Why haven't you heard anything?' and they were questions that we couldn't answer so it became easier for me to just not deal with anybody at all. I would probably say it was probably the worst moment of my entire life…* (**#50, mother of CF carrier)**.

During this period, some parents had felt depressed, been unable to sleep or concentrate at work, or described a negative impact on personal relationships.

*We had two massive blow ups at each other and it was a case of me...feeling left out that is what it was about really.* […] *you do feel that isolated, it's unbelievable. (**#41, Father of CF carrier; parents initially told ‘bad news' their child is a carrier, but may possibly have CF. The next day, at hospital, told child has CF but before diagnostic sweat test conducted. Subsequently, two sweat tests were negative for a diagnosis of CF.)***

Fathers also appeared to struggle because at the time of waiting for the second IRT result for CF many were back at work (The CF newborn screening protocol in England aims to identify a maximum of children with CF, while minimising carrier detection. This involves measuring immunoreactive trypsinogen (IRT) levels to identify babies with levels exceeding the 99.5 percentile, indicative of CF. Samples from newborns with raised IRT levels undergo DNA analysis to establish if the child has two mutations, and is therefore likely to have CF, or has one mutation and is probably a carrier. Children with suspected CF are referred for a clinical diagnosis. Those with one mutation, or an initial IRT >99.9 percentile and no detected mutation, require a second bloodspot test to verify if IRT is still elevated at 3–4 weeks when IRT levels are more discriminatory. In those children with one mutation, where the IRT remains elevated the likelihood of CF is regarded as high and triggers clinical referral, whereas if not still elevated the likelihood of CF is low and the child is regarded a healthy carrier.):

*I still went to work but I found myself hiding in my office more often than I normally would do.* […] *I found it very difficult to manage really just the day to day disciplining and just try to …. I don't know just try to keep it straight really.* (**#50, Father of CF carrier.)**

However further explanation and communication of results in discussion with a well-informed professional appeared to have allayed these concerns in most parents.

*Until I actually went to the* [genetics centre] *and that doctor probably explained it a lot more clearly, and to me it* [feeling of guilt]*sort of dampened down and from then on.. I could feel myself much more relieved by the whole sort of thing.***(#40, Mother of CF carrier; told needed second screening sample but was prewarned this is common and nothing to be concerned about. Was initially told child could be carrier, but not provided with further information at that stage by professional, so she sought information on internet and became upset)**

Only three of 38 parents of CF carriers were aware of their own CF carrier status before newborn screening of their child. In contrast, 21 of 29 parents of SC carriers were already familiar with their own carrier status through previous or antenatal testing and for these parents experience of distress appeared less common. Prior knowledge of personal carrier status appeared to help parents assimilate the information about their child *‘she is just like me, she is AC'* **(#3, mother of SC carrier)**.

Parents who had reported considerable distress regarding NBS in their original interview later felt, during respondent validation, that they had now come to terms with the experience, and continuing anxiety was rare. This was usually as a result of subsequent direct communication with a health professional. One participant reported continuing occasional concerns when their child, a carrier of CF, became unwell with a cold.

### Considering ‘cascade' testing within the family

For many parents, knowing their child's carrier status led them to question, and consider establishing, which parent was a carrier if this was not already known; and to consider the carrier status of their other children. In relation to parents' own carrier status, the issue of non-paternity was tangentially raised in three cases: parents' carrier status was seen to offer reassurance of paternity in two cases and in one case an oblique reference was made to the child's inheritance of a gene ‘skipping a generation'. Some parents had begun to worry if their other children had not been tested and were approaching reproductive age, or were from previous relationships, and how they might broach this with their children.

*It's just lingering because I have got two children from my previous marriage and if I am the carrier then it would be nice to inform them and tell them, you know, what would they like to do about it?***(#5, Father of a CF carrier.)**

While some parents felt unable to ‘move on' until testing had occurred for themselves or been offered to other relevant family members, others varied in their desire to do so, and how it may affect their future decisions.

*I am of the opinion of I don't want to* [know] *whereas you* [referring to father] *are more inclined that you do want to know.***(#19, Mother of a CF carrier.)**

Some parents, however, had experienced difficulties in accessing cascade testing.

*My other concern was before my 3½ year old was born they weren't specifically tested for this* [before universal newborn SC/CF screening] *so I wanted to know how I got him tested and [the health visitor] had no idea.***(#13, Mother of an SC carrier; sibling subsequently tested.)**

Others were concerned that despite their newborn being identified as a carrier, they were unable to access cascade testing for their other children until they were older (and had reached the age of consent themselves), encountering no flexibility in the system:

*When we told her* [sibling of newborn carrier] *we said, you know, they've said you need to have this test when you're 16 and she just turned round and went ‘Why can't I have it now?'***(#12, Mother of a newborn CF carrier, older sibling aged 10 years.)**

This was a particular source of parents' frustration:

*…they won't let me have her tested. The argument is she has to wait until she is 16 and classed as an adult or whatever it was* […] *but I feel that we are her parents and should be able to be responsible* (**#64, mother of CF carrier, sibling not tested)**.

### Sharing carrier information with extended family

Most respondents felt a responsibility to share their newborn's carrier status information with their extended families, but some struggled with knowing who to tell or how to raise the issue, or were concerned about creating anxiety.

*… my cousin's girlfriend was pregnant, and* [my partner] *was saying ‘You should tell them about the cystic fibrosis thing' and I was thinking ‘I shouldn't tell them about the cystic fibrosis thing because they've got 9 months of worry and then for them to think the baby will have an abnormality when its born' and I don't think that's fair.***(#37, Father of a CF carrier.)**

Others had no concerns about telling family members and discussed how their extended families appeared to respond positively to the news, by showing interest, getting tested and understanding that carrier status had minimal health implications.

[The whole family came] *round for Sunday lunch, we'll sit round the table, we'll discuss it. And it was really nice you know; you don't have to worry.***(#12, Mother of a CF carrier.)**

However respondents also reported negative reactions from their families including relatives avoiding related conversations; and refusing to believe the information in a context of stigma and ignorance about the conditions.

*..talked to my mother about it so many times, she doesn't want to listen, it's like* […] *she has turned a blind eye to it and I said to her that you have to get all my younger sisters to have them tested and she has turned a blind eye to it and so I have stopped talking about it.* (**#3, Mother of an SC carrier.)**

Family members also distanced themselves from the issue by blaming the other side of the family. When this occurred between parents it could exacerbate previously fractious relationships.

[the wider family] *were just looking at it as a negative, do you know what I mean? They were looking at it as if something that … like really ashamed and it was like ‘Oh none of us are affected'.* (**#37, Mother of a CF carrier.)**

Some families did not wish to pursue cascade testing creating ambivalence among parents of carriers who did not feel it appropriate to dictate to others, but who also had concerns that they should be more proactive to ensure this occurred. The lack of family members' engagement in testing could cause some distress.

*it came out that it was his family, but you know I have told him, but none of his family want to be tested even though his niece is trying to have a baby and… They don't really, yeah I begged her but she is not in their family and they don't want to be tested for it you know.* (**#22, Mother of a CF carrier.)**

Some felt health professionals should offer advice on how to discuss the issue or become involved in such communication. Where this had occurred, it was appreciated:

[the health professional] *was trying to be really helpful she e-mailed* [husbands' name] *the letter* [containing the carrier information] *so that he could get it to his family before and then she posted it out...***(#58, mother of CF carrier)**.

## Discussion

This study suggests that disclosure of CF or SC carrier results following universal newborn screening can have significant effects for parents and wider family members. For many parents, although being informed of their child's carrier status was positive, and this information was valued, there was considerable anxiety and distress following the initial communication of results. However, subsequently, parents appeared capable of understanding and assimilating this information into their lives. This suggests that concerns about provoking a vulnerable child syndrome^15^ may be avoided given appropriate communication and support.

The process of communicating the result, including messages from health professionals which contained insufficient information, or waiting for information, was not benign and was commonly reported as a key source of any distress, impacting social engagement. This echoes work conducted in the US and Australia in relation to CF newborn screening.^22, 23^ Our data also suggest sometimes wider detrimental impact on employment, parental relationships which may already be under some strain with the arrival of a newborn, and in particular, on the extended family. This adds to evidence that parents' information needs may not have been met in a timely or adequate manner and that parents may find newborn screening distressing.^14, 24, 25, 26, 27, 28, 29^ Ensuring that parents are not left in an information vacuum at any stage of the screening process may minimise the risk of harmful effects from screening.

Although some parents required additional specialist services, once carrier results were understood this knowledge did not appear to have a lasting negative effect on most parents in this study and was highly valued – as has been found previously in other countries.^20, 22, 30, 31^ Parents in the current study appear to underline this by viewing any change to protocols to not identify carriers as a backwards step. These findings can inform the ongoing debate about whether to identify carriers or inform parents,^32^ and conflicting findings regarding the impact of doing so.^13, 23, 30^ The results also address an identified gap in the evidence regarding communicating SC carrier rather than CF carrier results^33^ and whether it is true that the lack of repeat testing in SC screening means there is less anxiety.^20^ Although most of the distress in CF screening centred around repeat testing in the current study, we still found concerns and anxiety in those within the SC sample. The issue of involving fathers more in antenatal and neonatal screening has been raised previously^34^ and the current data highlight the disempowering effects on fathers when communication of results occurs solely with the mother. Although referred to, the issue of non-paternity being revealed by newborn carrier results did not feature prominently with parents interviewed in this study, though it is recognised that they were providing ‘public' accounts of their experiences to researchers.

The current findings start to address the paucity of data regarding how and what parents are told regarding NBS results.^35^ In doing so, they underline that communication of information and support following newborn screening is required beyond the infant period to facilitate access to cascade testing and support the challenges of familial communication. The issue of families avoiding communication about carrier information, and inferring blame or stigma has been discussed in relation to antenatal screening for sickle cell,^34^ as have the difficulties in working out who to tell and when.^34^ The current study highlights the same for newborn screening and the current lack of a defined role for any specific professional to provide such support. Given the period of time it may take for families to adapt to this information, this role might, for example, be developed in primary care, as part of continuing care with families, or within-community-based genetic counselling. Our results also highlight that parents' concerns regarding communication processes for receiving carrier results are nevertheless similar to parents who receive results that their child is affected by the condition.^35^ Moreover, we have found that these concerns, previously identified when screening is mandatory and information provision is assumed to be minimal, can also occur in a newborn screening programme that adopts an informed consent model.

### Strengths and limitations

A range of methods were used to enhance rigour, and the relevance of study results including purposeful sampling, iterative data generation, analysis to saturation, involving researchers from several disciplines and use of respondent validation.^36^ The sample is described in some detail including, for example, information on parents' own carrier status and social backgrounds, and the diversity of provider methods of communication of carrier results that they experienced in practice. This may aid assessment of the transferability and relevance of the findings beyond the immediate study context.

The sample is substantial when compared with previous qualitative work with parents of SC carrier,^37^ CF carriers^22, 30, 38, 39^ or both types of carrier.^40^ Those recruited also represent a significant proportion of the very small number of newborn CF carriers identified annually in England. Within the practical constraints of study recruitment and duration, the research has successfully included parents' experiences across all health regions of England following the introduction of universal newborn screening for SC and CF. The lack of fathers' perspectives in newborn screening research has caused concern,^41^ but the current study included fathers wherever possible, ultimately forming a quarter of the sample.

Although active inclusion of those willing to articulate their experiences is key to qualitative enquiry, we recognise that the experiences and views of study participants may not be typical of all receiving carrier results following newborn screening. Our sample was relatively well-educated, a feature similar to other studies concerning CF screening in particular^13, 42^ and appeared relatively genetically literate. Their experiences may however serve to reinforce the identified needs to improve information delivery and continuing support for the wide range of parents who experience newborn screening.

Further research should investigate how best to prepare parents for NBS before results as this appears to affect the impact of results on parents; and examine parents' attitudes towards, access to and experience of cascade testing and its impact on later reproductive decisions. In parallel, work is needed to develop and evaluate methods to support cascade testing and communication of carrier information with children and families; and capture further experience of families over time to enable greater understanding of longer-term benefits or harm of newborn carrier identification.

## Figures and Tables

**Table 1:** Characteristics of parent respondents^a^

*N=67 (%)*	
*Ethnicity*	
White British	43 (64)
Black African/British/Caribbean	14 (21)
Bangladeshi	3 (4)
White European/Other	3 (4)
Mixed	3 (4)
Asian Thai	1 (1)
	
*Employment status*	
Employed full time	33 (49)
Employed part time	12 (18)
Full-time parent	11 (16)
Full-time student	3 (4)
Full-time carer	1 (1)
Unemployed	1 (1)
Not indicated	6 (9)
	
*Highest educational attainment*	
Degree or higher degree	22 (33)
NVQ, Diploma, A-level, HND or equivalent	17 (25)
GCSE or equivalent (school to 16 years)	14 (21)
None	1 (1)
Not indicated	13 (19)
	
*Parent carrier status*	
Not tested	19 (28)
Tested – carrier SC	17 (25)
Tested – carrier CF	11 (16)
Tested – not carrier CF	11 (16)
Tested – not carrier SC	9 (13)

a Includes two grandparents.

**Table 2:** Characteristics of newborn infants, *N* (%)

*N=51*	
* Infant carrier characteristics*	
* *Carrier of CF	27 (53)
* *Carrier of SC/other haemoglobin variant	24 (47)
	
* Gender*	
* *Female	24 (47)
* *Male	27 (53)
	
* Family units*	
* *Mother lives with baby's father	43 (84)
* *Mother lives with partner	1 (2)
* *Single parent household	7 (14)
