# Supplementary material for: Parents' responses to receiving sickle cell or cystic fibrosis carrier results for their child following newborn screening
Source: Eur J Hum Genet. 2014 Jul 9;23(4):459–65. doi: 10.1038/ejhg.2014.126 (PMC4666569; doi:10.1038/ejhg.2014.126)
Supplement: Supplementary Information [file ejhg2014126x1.doc]

**SUPPLEMENTARY INFORMATION**

**Table 3: Communication of SC carrier results experienced by parent sample**

| **Communication of SC carrier results experienced** | **Parents [n=29]**  (high / low SC prevalence area) |
| --- | --- |
|  |  |
| **Haemoglobinopathy Counsellor** | **14 (11 high /3 low)** |
| *Letter* (result)*, health centre consultation* offered and taken up | 6 (4 high /2 low) |
| *Letter* offering appointment, *health centre consultation* (result) | 5 (high) |
| *Letter* alone (result) | 2 (high) |
| *Phone call* (result), advised to consult GP, second *phone call* from haemoglobinopathy counsellor | 1 (low) |
| **Health Visitor** | **7 (2 high /5 low)** |
| *Home visit* (result) | 3 (low) |
| *Home visit* (result), further *home visit* from specialist screening counsellor | 1 (low) |
| *Home visit* (result), GP visit, *phone call* from haemoglobinopathy counsellor | 1 (low) |
| *Phone call* (result) | 1 (high) |
| *Phone call* (result), *home visit* | 1 (high) |
| **Screening Link Health Visitor** | **4 (1 high /3 low)** |
| *Letter* (result), *home visit* with family health visitor | 2 (low) |
| *Phone call* (result), *home visit*) | 1 (low) |
| *Phone call* (result) | 1 (high) |
| **Specialist Screening Counsellor** | **3 (low)** |
| *Letter* (result), *home visit* | 3 (low) |
| **Midwife** | **1 (low)** |
| *Home visit* (result), GP consultation | 1 ( low) |

**Table 4: Communication of CF carrier results experienced by parent sample**

| **Communicating need for/**  **performing second heel prick test** | **Communication of CF carrier result** | **Parents**  **(n=38)** |
| --- | --- | --- |
|  | **Specialist screening nurse** | **12** |
| Midwife-*phone call, home visit* | Specialist screening nurse-*phone call, home visit* (result) | 5 |
| Midwife-*home visit* | Specialist screening nurse-*phone call, home visit* (result) | 2 |
| Specialist screening nurse-*phone call,* health visitor-*home visit* | Specialist screening nurse-*phone call, home visit* (result) | 2 |
| Specialist screening nurse-*phone call,* midwife,-*home visit* | Specialist screening nurse-*phone call, home visit* (result) | 1 |
| GP-*phone call,* midwife-*home visit* | GP consultation (result), specialist screening nurse-*phone call & letter* | 1 |

| Interviewee cannot recall | Specialist screening nurse-*phone call, home visit* (result) | 1 |
| --- | --- | --- |
|  | **CF specialist nurse** | **6** |
| Midwife-*home visit* | CF specialist nurse-*home visit* (result) | 2 |
| Midwife-*home visit* | CF specialist nurse-*hospital consultation* (result) | 1 |
| Midwife-*answer phone, home visit* | CF specialist nurse & Consultant-*hospital consultation* (result) | 1 |
| No second test taken[[1]](#footnote-2)1 | CF specialist nurse-*home visit* (result) | 1 |
| Health Visitor-*home visit* | CF specialist nurse-*phone call* (result) | 1 |
|  | **Hospital Consultant** | **6** |
| GP-*during consultation,* CF nurse & health visitor-*home visit* | Hospital consultant clinic (result) | 2 |
| Midwife-*phone call, home visit* | Hospital consultant clinic (result) | 2 |
| Midwife-*home visit* | Health visitor (child carries one gene), hospital consultant (incorrectly informed child affected, before confirming is carrier) | 2 |
|  | **Specialist counsellor** | **5** |
| Midwife-*phone call, home visit* | Specialist counsellor-*phone call* (result), *home visit* | 2 |
| Midwife-*phone call, home visit* | Specialist counsellor-*phone call* (result), specialist counsellor & health visitor-*home visit* | 2 |
| Interviewee cannot recall | Health professional (unclear who)-*phone call* (result), specialist counsellor-*home visit* | 1 |
|  | **CF nurse & ‘familiar’ health professional** | **4** |
| Midwife-*phone call, home visit* | GP-*phone call* (result), CF nurse & health visitor-*home visit* | 2 |
| Midwife-*home visit* | CF nurse & midwife-*home visit* (result) | 2 |
|  | **Genetic counsellor** | **3** |
| Midwife-*phone call, home visit* | Genetic counsellor-*phone call* (result), *hospital consultation* offered but not accepted | 1 |
| Midwife-*home visit* | Genetic counsellor-*phone call* (result), *hospital consultation* offered and taken up | 2 |
|  | **Screening Midwife** | **1** |
| Midwife-*phone call, home visit* | Screening Midwife-*phone call* (result) | 1 |
|  | **Screening link health visitor** | **1** |
| Health visitor – *phone call, home visit* | Screening link health visitor – *phone call* (result) *home visit* | 1 |

1. ^^ risk of CF identified antenatally, sweat test conducted [↑](#footnote-ref-2)
